# Pharmacologia; Being an Extended Inquiry into the Operation of Medicinal Bodies, upon Which Are Founded the Theory and Art of Prescribing

**Published:** 1843-10-01

**Authors:** 


### Pharmacologia; being an extended Enquiry into the Operation of Medicinal Bodies, upon which are founded the Theory and Art of Prescribing. By *J. A. Paris*, M.D. Edition Ninth. London: Highley, 1843.

[*Concluded from No. LXXVII. p.* 34.]

We are now come to that division of his subject called by our author Local (or Special) Stimulants.

This, his second division, comprehends those medicinal agents which have been classed under the head of *Evacuants*.

The propriety of giving the name of stimulants to substances which, by their evacuating properties, lower the system so very much as we know emetics and purgatives do, is certainly more than questionable. The effect of a dose of senna and salts can scarcely be said to be of a stimulating character—however, as the term is here understood conventionally, transeat.

*Emetics.*—Our author defines emetics to be " substances which excite vomiting, independent of any effect arising from the stimulus of quantity, or from that occasioned by any nauseous taste or flavour."

The act of vomiting was formerly attributed to the sole influence of the stomach. Majendie, however, will have it, that the act of vomiting is attributable, exclusively, to the agency of the brain on the abdominal muscles, considering the stomach to be a mere passive instrument on the occasion. Those even who may not be disposed to admit Majendie's theory in its full extent, are willing to admit that the influence of the nervous system is indispensably necessary for producing vomiting; and accordingly we find that, where the energy of the nervous system is suspended, vomiting will not take place, however forcibly the stomach may be goaded by emetics.

"Vomiting may also be produced by the *primary* operation of certain agents upon the brain, by which its energy is disturbed, as by narcotics, or by the motions of swinging, whirling, or sailing; in such cases, the series of actions necessary for the establishing of vomiting, commences in the brain, and is propagated by nervous sympathy to the stomach."

With respect to the mechanism of vomiting, Dr. Marshall Hall considers that, during the act, the larynx is closed, the diaphragm and its various apertures relaxed, while all the muscles *of expiration* are called into action, and yet actual expiration is prevented by the closure of the larynx; in consequence of this, the spasmodic effort expends all its force upon the stomach, and since the cardiac orifice remains open, from the relaxed state of the diaphragm, vomiting takes place—whatever theory be adopted the necessity of nervous influence remains undisputed.

"When an emetic is taken into the stomach, an interval of twenty minutes, or longer, usually passes without any apparent effect, a fact which certainly favours the idea of absorption; although it may also be immediately induced by mechanical irritation of the pharynx, stomach, or intestines, which proves that it is effected through the medium of the nerves. An uneasy sensation, which we term nausea is then felt, and this continues to increase, until vomiting begins. Here then we perceive are two distinct stages, each of which is marked by its own proper symptoms: the relative intensity and duration of which will be found to vary according to the nature of the exciting causes; thus some emetics, as sulphate of zinc, act without occasioning much nausea, while others, as *tobacco*, excite it to a degree, which is far greater than is proportioned to their emetic power; this is a fact of great importance in directing us in the selection of an emetic, for we shall find that in some diseases it is a great object to avoid that state of system which invariably accompanies nausea, while in others it affords the best mode of answering an important indication of cure."

During the operation of an ordinary emetic, the following are the symptoms which characterise the two stages; while the nausea only is present, the countenance is pale and shrunken, the pulse feeble, quick and irregular, and there is a feeling of cold; but as soon as vomiting commences, the face becomes flushed, the pulse quicker and stronger, although it seldom returns to its natural standard, until some time after the vomiting has ceased. A degree of languor, a disposition to sleep, and a general moisture upon the skin, are the circumstances which occur after the total cessation of the paroxysm.

The advantages to be derived from the employment of an emetic in the treatment of disease may depend either on its *primary* or *secondary* effect, that is either on the mere evacuation of the stomach, or on the changes in remote parts arising from sympathy. In selecting the emetic the practitioner should be directed by the object he wishes to fulfil. If he merely wish to evacuate the stomach, he will avoid nauseating emetics and select one that will act quickly. If, on the contrary, he wish to affect some remote organ through the medium of sympathy, an emetic of an opposite character should be preferred. In some cases the mechanical action of the diaphragm and abdominal muscles is the object aimed at; jaundice, arising from the obstruction of biliary calculi, has frequently been quickly removed by the operation of an emetic.

Among the secondary effects of emetics may be reckoned their abating the force of the circulation from the nausea which they induce; hence their use in the treatment of hæmorrhage—in cases of menorrhagia, ipecacuanha has been found of decided benefit. From the function of absorption being inversely as the force of the circulation, we derive considerable aid in the treatment of anasarca from the use of nauseating emetics. Emetics may also be useful in relieving congestion of the capillaries. When we wish to facilitate the passage of a gall-stone through the ductus communis choledochus, a *nauseating emetic* should be selected from the well known efficacy of nausea in relaxing, while the mechanical concussion tends to drive the obstructing matter forward. When on the contrary we wish to evacuate the stomach and to prevent absorption, we should by all means avoid a nauseating emetic; this should be carefully attended to in cases of poisoning. Diaphoresis is frequently observed to follow vomiting, the vessels of the skin sympathising with the stomach.

It may be inferred *à priori* that vomiting may not be devoid of danger in certain states of the body. From the pressure applied to the descending aorta and the interrupted circulation through the lungs and from impeded respiration, the blood returns with difficulty from the head during vomiting—in the advanced stages of pregnancy the concussion of an emetic may be mischievous, as also in *hernia* and *prolapsus uteri*—in extreme debility also the violence of an emetic may produce fatal syncope.

Jaundice is frequently induced by long continued retching. This arises from the great irritability of the diaphragm and abdominal muscles occasioned by the violent and repeated efforts to vomit; these muscles are thereby thrown into strong spasmodic contractions, and the liver, with the gall-bladder, will be suddenly caught, and as it were squeezed in a press, whereby the bile is made to regurgitate and is carried into the *venæ cavæ*. Such a jaundice will disappear without the aid of any medicine.

*Cathartics.*—These are defined to be medicines which quicken or increase the evacuation from the intestines, or which, when given in a certain dose, occasion purging. They are divided into *laxatives* and *purgatives*. The former merely evacuate the contents of the bowels without at all affecting the exhalent vessels of the canal; the latter produce a considerable influx of fluids from these vessels; when the purgative is very violent, it is termed *drastic*.

The effects of a purgative may depend on three different modes of operation, viz.

1. By stimulating the muscular fibres of the intestines, whence their peristaltic motion is augmented, and the contents of the bowels more quickly and completely discharged.

2. By stimulating the exhalent vessels terminating in the inner coat of the intestines, and the mouths of the excretory ducts of the mucous glands; by which an increased flow of serous fluids takes place from the former, and a more copious discharge of mucus from the latter; the effect of which is to render the fæcal matter thinner and more abundant.

3. By stimulating the neighbouring viscera, as the liver and pancreas, so as to produce a more copious flow of their secretions into the intestines.

Different purgatives are well known to possess different powers in relation to their several modes of operation; some medicines, as manna, sulphur, magnesia, urge the bowels to evacuate their contents by an imperceptible action on the muscular fibres, and scarcely any increase of serous discharge; others seem to increase the peristaltic motions by acting as mechanical stimulants on the fibre. Other cathartics stimulate the fibres still more, and the effects are either confined to some part of the canal, or communicated to the whole of the intestines from the duodenum to the extremity of the rectum; aloes is an example of the former, and colocynth of the latter mode of operation. Other cathartics again act on the exhalants and produce serous evacuations, and hence formerly called *hydragogues*; these are generally *saline bodies*. *Mercurial purgatives* chiefly act on the liver and thereby occasion an influx of bile into the intestines. The value of purgatives in very many diseases is very great; in none, however, is it more necessary to stimulate the intestines to evacuate their contents than in fever, where, from the oppressed state of the nervous system, the peristaltic motion of the intestinal tube is considerably impaired and fæcal accumulations are sure to take place. We may remark of purgatives (and the same remark applies to other classes of medicines), that there is less tolerance of them in health than in the state of disease for which they are indicated. A healthy person will have his bowels opened by touching the tongue with the cork of a croton oil bottle, whereas it will require perhaps two or three drops to purge a person who is feverish. It is well known what large doses of tartar emetic may be tolerated in inflammatory diseases, and how little in health, or in chronic affections; as also what large doses of brandy and opium are required to produce an effect in delirium tremens.

In certain of the neuroses connected with a morbid condition of the alimentary passages, purgatives are essentially serviceable; thus *chorea* and *hysteria* have been very successfully treated in this way—the same may be said of *chlorosis*. Nor are the beneficial results of purgatives confined to the mere feculent evacuations which they may occasion. By the quantity of serous fluid which they abstract from the current of the circulation, they are useful as *antiphlogistics*. Where it is an object to promote absorption, they are of great use; according to the well known law that absorption varies directly as the exhalation. Mercurial purgatives not only evacuate the bowels, but also act on the neighbouring organs, and remove congested states of the liver and ducts.

The principle that different purgatives exert their action on different parts of the tube, and possess distinct powers over the different species of matter to be evacuated, should not be lost sight of by the judicious practitioner. Perhaps there is no class of medicines more abused than the class now under consideration. Where there is great debility of constitution great caution is necessary in prescribing the more active remedies of this class, and also where there is any tendency to gastric or intestinal inflammation. Delicate subjects, whose nervous systems are very weak, find it necessary from the effect of habit to have frequent recourse to purgatives; a free use of tonics, however, and more especially of the preparations of iron, will, by strengthening the nervous system, prove a much better and more certain mode of keeping the bowels open. During pregnancy, and immediately after delivery, and also during menstruation, caution is required in the administration of purgatives.

*Emmenagogues.*—These are medicines which are capable of producing the menstrual discharge.

That emmenagogues are but relative agents will appear evident if we only recollect the various morbid states on which retention or suppression of the menses may depend.

This affection generally depends on debility of the system; it sometimes however depends on, or at least accompanies, a plethoric habit of body; in the former case tonics are indicated, in the latter, venæsection aided by purgatives, will act the part of an emmenagogue; cupping on the loins has often been found very serviceable. When the affection occurs in young women about the age of puberty, it is generally connected with extreme debility, and the preparations of iron, bark, and other tonics, are the most likely to succeed; but in full, florid habits, where the catamenia are suddenly suppressed, antiphlogistic means, such as gentle purgatives, blood-letting, &c. are the most likely to prove serviceable.

There are two other classes of medicines, which are sometimes found beneficial in treating amenorrhœa, viz. *acrid purgatives* and *stimulating diuretics*; the former act on the rectum and thence by contiguous sympathy on the uterus, as *aloes*, &c.; the latter, as *cantharides*, the *turpentines*, are supposed to excite the womb sympathetically by their stimulus on the bladder—a combination of myrrh, aloes, sulphate of iron and the essential oil of savine has been found very useful. Mercury also has been found of great service.

*Diuretics.*—In this section, by far the most valuable and practically useful in the entire work, the action and modus operandi of diuretic medicines, or medicines which increase the urinary discharge, is considered. The following classification of these substances is proposed by our author:

Class I. Medicines which act primarily on the Urinary Organs.

1. *By stimulating the Secreting Vessels of the Kidneys, by Contact.*

*a.* The medicines not undergoing decomposition *in transitu.*

1. *Potassa.*

2. *Potassæ Nitras.*

3. *Oleum Terebinthinæ.*

4. *Juniperus Communis.*

5. *Cantharides.*

6. *Potassæ Hydriodas.*

*b.* The medicines undergoing decomposition *in transitu.*

1. *Potassæ Acetas.*
2. *Potassæ Bitartras.*
3. *Scilla Maritima.*
4. *Colchicum Autumnale.*
5. *Copaifera Langsdorfii.*
6. *Cytisus Scoparius.*

Class II. Medicines which act *primarily* on the Absorbents, and *secondarily* on the Kidneys.

*Mercury.*

Class III. Medicines which act *primarily* on the Stomach and Primæ Viæ, and *secondarily* on the Absorbents.

1. *By diminishing Arterial Action, and increasing that of Absorption.*

1. *Digitalis.*
2. *Nicotiana Tabacum.*

2. *By increasing the Tone of the Body in general, and that of the Absorbent System in particular.*

*Bitter Tonics, &c. &c.*

3. *By producing Catharsis, and thereby increasing the Action of the Exhalants* directly, *and that of the Absorbents* indirectly.

1. *Elaterium.*
2. *Jalap, &c.*

It is easy enough to conceive that a substance capable of entering the current of the circulation, and of stimulating the kidneys by actual contact, should occasion a more copious urinary discharge; saline substances seem to be the peculiar stimuli of these organs, as potass, soda, nitre, &c. Water, as a simple diluent, will promote the action of the kidneys, and

Cullen remarks that, by withholding the use of fluids in dropsy, you diminish the quantity of fluids secreted. This will be sufficient answer for those who prohibit the use of all drinks in dropsy.

"It is observed that the digestive organs appear to possess the power of readily decomposing all saline compounds into which *vegetable* acids enter as ingredients, and of eliminating their alkaline base, which, being in the course of the circulation carried to the kidneys, excites them into action, and promotes the excretion of urine; and it is probably in this way that the *acetate, citrate, bitartrate,* and other analogous combinations of *potass* and *soda,* prove diuretic: on the other hand, it is equally evident that salts containing the *mineral* acids are not under the control of the decomposing powers of the *chylopoietic* organs, and consequently do not undergo any changes *in transitu,* although some of these salts, as I have just stated, especially the more soluble ones, are absorbed entire, and prove diuretic. *Sulphate of potass,* from its insolubility, is not readily absorbed, and its composition will not allow the development of its base; we perceive, therefore, that it has not any tendency to produce an influence upon the urinary secretion."

Certain vegetable bodies likewise appear to occasion diuresis by a similar mode of operation, and it is worthy of notice that these medicines generally contain a bitter principle, which is probably separated by the analysing powers of the stomach, as exemplified in the *scilla maritima, colchicum autumnale, lactuca virosa, gratiola officinalis, cytisus scoparius* (*cucuminas*), *juniperus communis,* &c. The stimulant powers of a bitter vegetable principle upon the *primæ viæ* we have already noticed under the head of Tonics. Our author makes a remark here well deserving the attention of the practitioner, viz. that the diuretic operation of any body that acts by being absorbed, is at once suspended, if catharsis follows its administration, whether in consequence of the largeness of its dose, its increased solubility, or from the effect of its combination with some purgative, for it is a law, that the processes of assimilation and absorption from the duodenum are arrested, or very imperfectly performed during any alvine excitement; cream of tartar is a good example of this, which, in moderate doses, acts on the kidneys; but if the dose be increased, or if it be reduced to the state of tartrate (*soluble tartar*), so that catharsis follows its employment, then diuresis will not ensue, since no decomposition can take place under such circumstances, nor can it be carried by absorption into the circulation. Oil of turpentine is another instance of the same law; in the dose of two fluid drachms it will act powerfully on the kidneys, whilst in the dose of a fluid ounce it will scarcely exercise any influence on these organs, from the increased dose acting on the bowels, and consequently preventing its entrance into the circulation.

Another fact is mentioned by our author, illustrative of the same principle, viz. that sulphate of magnesia will not readily produce diuresis, because it acts on the bowels; but if this same saline be given to the horse, whose bowels are not readily affected by purgatives, it acts powerfully on the kidneys; from this in-irritability of the bowels of the horse, diuretic medicines are more certain in their operation on that animal than in the case of the human subject; a fact which, in itself, as the author very happily remarks, shows the importance of attending to the bowels, during a course of those diuretics, which require to be absorbed before they can produce their specific effects.

The state of the cutaneous surface demands attention during the administration of a diuretic; for, if its vessels be excited by external warmth, the action of the diuretic may be diverted from the urinary organs to the exhalants on the surface, and occasion diaphoresis; whilst, if the surface be kept cool, this diversion will not occur; so effectual, in fact, is cooling the surface in determining to the kidneys, that the ordinary diaphoretic medicines may, by an attention to this circumstance, be converted into powerful diuretics, from the close sympathy subsisting between the functions of the skin and kidneys.

The second class of medicines established by our author includes those which act *primarily* on the absorbents, and *secondarily* on the kidneys. We have already seen, that, by increasing the action of the kidneys, we also augment that of the absorbents. Here we bring about an operation the very converse of that; the absorbents are stimulated in the first instance, the blood then becomes surcharged with serous fluid, in consequence of which the kidneys are called into brisk action, and this serous fluid is eliminated through the urinary passages. The only substances with which we are acquainted as specific stimulants of the absorbents are mercury and iodine.

Class III., we have already seen, includes those substances which act *primarily* on the stomach or system, and *secondarily* on the urinary organs.

1st. By diminishing arterial action, and increasing that of absorption.

It is an established truth in physiology, that the energy of the vessels circulating the blood and that of the absorbents are antagonist powers. Majendie has proved, that the absorption of a poison is retarded by a plethoric and accelerated by a depleted state of the blood-vessels. Hence, from the close connexion which exists between arterial action and absorption, it is evident that medicines lowering the former, must augment the latter, and increase diuresis. Hence the value of digitalis in the treatment of dropsy; its powers in augmenting diuresis appear only when it is given in dropsy. For the same reason, and on the same principle, we know venæsection proves a most effectual diuretic.

2. By increasing the tone of the body in general, and that of the absorbent system in particular.

We frequently observe that fevers which have been protracted are followed by *œdematous* swellings; this we explain by the general debility of the body, in which the absorbents participate; the indication in such cases is to invigorate the system, whereby the enfeebled absorbents are strengthened and stimulated to take up and remove the accumulations of serous fluids. In extreme debility, as well as weakness of the absorbents, it would seem that we have laxity also of the exhalants, which causes these vessels to allow the thinner parts of the blood to pass too readily through them; there are many facts which tend to prove the truth of this mode of viewing the subject; palsied limbs frequently become œdematous; the advantage resulting from the mechanical support of pressure from bandages in such cases is another proof.

3. By producing catharsis, and thereby increasing the action of the exhalants *directly*, and that of the absorbents *indirectly*.

Our author has already noticed under the head of Cathartics that these medicines are capable of causing a very copious discharge of serous fluid; by this the blood is deprived of a large portion of water, and the absorbents are thus indirectly stimulated to supply the deficiency. Elaterium and jalap are the principal substances employed for this purpose—where the vital powers are adequate to sustain the violence of these remedies, especially of the elaterium, they are strikingly effectual in several forms of dropsy. We have now presented a rather full analysis of our author's observations on the employment of Diuretics in the practice of medicine; they are extremely valuable; for they lead the practitioner to select the particular diuretic best calculated to fulfil the indications of each individual case; they will at the same time point out those which are physiologically incompatible. Where dropsy arises from organic disease, its cure cannot be accomplished by the mere evacuation of the fluid, but where it arises from diminished absorption, then it is that diuretics prove most effectual. We now come to those medicines which increase the natural exhalation of the skin, and which have been called *Diaphoretics* or *Sudorifics*, according to the degree of force with which they act; in the one case the effused fluid is carried off by the conducting power of the atmosphere, in the insensible form of vapour, whilst in the other it is effused so copiously from the exhalant vessels, as to appear in the liquid form. The following is the arrangement proposed by our author of the medicines of this class—

DIAPHORETICS produce their effects—

I. BY STIMULATING THE CUTANEOUS CAPILLARIES.

A. By external application.

*By stimulus of heat, friction, &c.*

B. By medicines which enter the circulation, and stimulate the cutaneous vessels by contact.

*Mercurials—Sulphur.*

C. By medicines which act on the surface *sympathetically*, through the medium of the stomach.

*Cold Drinks, &c.*

II. BY INCREASING THE GENERAL ACTION OF THE VASCULAR SYSTEM.

*Violent Exercise—Ammonia—Guaiacum—Alcohol—Warm Bath.*

III. BY RELAXING THE MORBIDLY CONSTRICTED MOUTHS OF THE PERSPIRATORY VESSELS.

*Antimonials—Cold Affusion—Venæsection—Saline Diaphoretics.*

Heat alone is often sufficient to produce diaphoresis, without necessarily increasing that of the heart and arteries; it generally too accelerates the operation of a sudorific medicine; there are occasions however, where the stimulus of heat will be found to impede diaphoresis; as in the hot stage of continued fever, where there seems to exist a peculiar constriction of the perspiratory vessels, accompanied with heat and dryness; in which case, remedies of the third class should be employed. The *warm bath* seems to partake of *all* the qualities upon which the above classification is founded. During the ardent heat of fever the external application of cold is the most efficient sudorific—cold drinks also are an excellent diaphoretic in the hot stage of fever; this effect they produce through the sympathy subsisting between the stomach and skin. In the same way is explained the diaphoresis occasioned by the use of nauseating doses of antimony, and of other emetics.

Those stimulants which are found to produce sweating do so by merely accelerating the circulation; great care is necessary in the employment of the substances; especially in febrile affections.

*Saline Diaphoretics* are supposed to enter the circulation and thus to act directly on the cutaneous vessels.

*In treating pulmonary affections*, diaphoretics are found very useful from the sympathetic connexion subsisting between the functions of the lungs and skin. In bowel complaints also they are employed with advantage for a somewhat similar reason. We know that sudden suppression of perspiration by cold is frequently followed by *diarrhœa, enteritis, &c.* These medicines are often employed in *some skin diseases*, as *herpes, lepra, &c.* In the various forms of *dropsy*, especially anasarca, diaphoretics are useful.

It is known that by the cutaneous transpiration a portion of excrementitious matter is removed from the system; if therefore this function be but imperfectly performed, a deleterious fluid is retained which may occasion disease; such a course our author suspects may have some share in the generation of calculi, and other diseases of the urinary organs.

The next class of medicines which presents itself includes Expectorants, or substances supposed capable of facilitating the excretion of mucus from the breast, viz. from the trachea, and cells and passages of the lungs. The author however repudiates the idea that there are any substances which can effect this by any *specific action* on the parts concerned.

In order to explain their *modus operandi* he proposes the following classification of them, founded on their supposed modes of operation:—

Class I.—Medicines which increase Pulmonary Exhalation, and thereby dilute the Mucus in the Follicles of the Lungs.

*a.* By removing constriction of the pulmonary exhalant vessels.

*Blisters—Venæsection—Nauseants.*

*b.* By stimulating these vessels by the *actual contact* of a medicinal substance.

*Allium—Scilla? Balsams—Fœtid Gums.*

*c.* By stimulating the top of the trachea, and thereby increasing the action of the exhalant vessels of the lungs, by a species of *contiguous sympathy*.

*Stimulating Lozenges—Linctusses—Inhalation of certain Vapours.*

Class II.—Medicines which diminish the inordinate flow of fluid into the Lungs, and render the Expectoration of the remainder more easy.

*a.* By removing the debility of the exhalants.
*Sulphate of Zinc—Bitter Tonics.*

*b.* By increasing the power of the absorbents.
*Digitalis—Nicotiana Tabacum.*

*c.* By determining to the skin by gentle diaphoresis.
*Antimonii Potassio-Tartras.*

*d.* By exciting serous discharges from the bowels.
*Saline Purgatives.*

Class III.—Medicines which operate mechanically, in promoting the Rejection of accumulated Mucus.

*a.* By stimulating the muscles of respiration.

*Ammonia.*

*b.* By exciting vomiting, and thereby compressing the thoracic viscera.

*Emetics.*

Class I.—The exhalants of the lungs, may, as well as those of the skin, be spasmodically constricted, and thus the usual quantity of fluid is not effused; in such cases, bleeding, blistering and nauseants are useful. Some substances which enter the circulation, are more particularly determined to the pulmonary vessels: *garlic* is an instance. Such substances may stimulate the exhalants through which they pass, cause them to pour out more fluid and so to dilute the viscid mucus, and facilitate its escape.

In persons debilitated by age or disease the exhalants of the lungs lose their tone and pour out too large a quantity of fluid; this is observable in *humoral asthma* and *catarrhus senilis.* This may be remedied by general tonics, and by local astringents of the exhalants. The author has found sulphate of zinc useful for this purpose.

The pulmonary absorption is promoted by small doses of mercury, *digitalis* and *nicotiana tabacum.*

In the act of vomiting the thoracic viscera are violently compressed, and the expulsion of mucus from the cavity of the lungs is thus promoted by the associated action of contiguous parts.

Atmospheric changes with respect to moisture and dryness are well deserving of the practitioner's attention; some excellent remarks are made here on their influence in expectoration; for which we refer to the book itself.

### Chemical Remedies.

There was a time, when the various functions of the body were regarded as processes wholly chemical,—when diseases were referred to chemical agency; and when, as a necessary consequence, medicines were only appreciated as chemical agents, calculated to control or correct such specific action. Those who maintained the supreme and exclusive agency of chemistry, were succeeded by others who denied its powers altogether, and affirmed, that no chemical change could ever take place in the living body, nor any chemical agent be brought to bear on an animated structure.

This controversy, however, like a great many others, in medical history, is in a great degree *verbal;* the ideas attached to the term *chemical,* not having been sufficiently distinct and definite, and each writer having so far adopted the definition, so as to suit his own preconceived opinions and views.

"It is evident," says our author, "that an organized body cannot generate matter, but can only change the modes of its combination, and that its sustenance and reproduction must depend upon the chemical transformation of the substances which are employed as its nutriment, and which contain its own elements. It is at the same time admitted that the attractions which take place under the influence of the vital principle, are generally essentially different from those, by which the same elements are actuated in the laboratory of the chemist; and hence we recognize the existence of a distinct set of laws, determining and regulating the combination and decomposition of all bodies appertaining to living structures, to which the general title of *Vital Affinities* has been applied; but the combinations having been thus determined, chemistry furnishes no farther analogies. The form which matter subsequently assumes is entirely due to vital power, which has nothing in common with physical agency; how, for instance, can we explain, as Liebig observes, its tendency to take the form of globules, or rather of cells, in which a containing cyst and a contained matter are usually discernible? We may, by chemical agency, produce the constituents; but we can never mould them into an organized tissue, or an organic cell.

"In order to avoid any misapprehension that may arise from the doubtful import of terms, the reader is desired to keep in mind the following definitions:—Chemical Changes are such as take place between the constituent parts of inanimate bodies, according to certain laws of affinity, which have been established by experiment, or deduced from theory. Chemico-Vital Changes are those transformations, or changes of composition, which result during the performance of the animal functions, but which, at least as far as the present state of our knowledge will enable us to judge, are not conformable with the laws which determine the combination and decompositions of inert matter."

The author remarks, that he does not pretend to say that these distinctions have any existence in nature; they merely mark the boundary of our present knowledge, and may change as science progresses; in fact, this imaginary line of demarcation has, within the last few years, so far altered its range as to have included within the limits of the first class (Chemical) several of the phenomena, which had long been regarded as belonging to the second, or Chemico-vital; as examples might be mentioned, the formation of *urea,* and the *formic, oxalic,* and *allantoic* acids, in our laboratories.

Professor Liebig, by applying to chemical action the dynamic law of La Place and Berthollet, viz. *that a molecule set in motion by any power can impart its own motion to another molecule with which it may come in contact,* has not only beautifully explained the phenomena of fermentation, putrefaction and decay, but, by showing that a body *in the act of decomposition* may, as it were, by an influence exerted beyond the sphere of its own attractions, impart its peculiar state of transformation to compounds with which it may happen to communicate, has gone far to furnish a parallel to the hitherto mysterious process of assimilation, while it has thrown considerable light on the action of certain organic poisons.

"As a general proposition we are justified in regarding vitality as a power engaged in continual conflict with the physical, chemical, and mechanical forms to which every species of inanimate matter is invariably subjected. The animal machine is constantly surrounded and assailed by agents whose elective attractions for the several principles of which it consists are so numerous and energetic, that its decomposition must inevitably and speedily result, were not the cohesion of its molecules, maintained by the conservative influence of a superior power—that power is LIFE; and as its energies decline we discover the ascendancy of chemical forces, until, at its final extinction, the elements of which the animal body consists fly off in the form of binary compounds, or, in other words, they undergo that series of changes which constitutes the phenomena of decay and putrefaction."

It was essentially necessary for the preservation of the body that it should have been endowed with a power of resistance to forces calculated to destroy it; it does not follow, however, that Nature will refuse the aid of such chemical powers as may assist her operations, or counteract their tendencies to error. "It would be truly extraordinary," says Liebig, "if the vital principle, which was everything for its own purpose, had allowed no share to chemical forces which stand so freely at its disposal." In fact, the animal economy, in a state of health, affords several obvious instances of chemical agency. In all the chemical processes which take place in the animal economy, we still perceive the control of a power, distinct from those agencies which connect themselves with the phenomena of inert matter.

Since, then, the vital principle, while it vigilantly guards the animal structure from such forces as may injure it, does not oppose such chemical agencies as may be salutary, we are warranted in arguing how far a medicinal substance may be brought to act *chemically* upon the living body.

Our author having shewn that chemical agency is not incompatible with the vital power, and how chemical action is controlled by the vital influence; having shown also the fallacies which the mere chemist is liable to fall into when he turns physician, then comes to consider the action of medicines as *chemical agents*, which subject he discusses under the four following heads:—

1. Can we by chemical means supply the living body with such materials as may be deficient?

2. How far are we able to neutralize, annul, or remove offending materials by chemical agency?

3. Can we oppose and counteract, by chemical means, any undue ascendency of chemical forces in the living body?

4. Are there any agents by which we can, chemically, influence animal temperature?

For the discussion of these questions we beg to refer the reader to the work itself, where he will find them very clearly and satisfactorily treated under the several heads to which they belong. We next come to the subject of—

Refrigerants, or Substances, which diminish the force of the circulation, and reduce the morbid heat of the body, without occasioning any diminution of sensibility or nervous energy. These may be external or local, or internal and general. In the first case their claims to chemical agency are clear enough; not equally so, however, in the latter.

*Topical Refrigerants.*—In cases of external inflammation, refrigeration may be produced by cold applications, cold water, ice, certain saline bodies in solution, or by evaporation, which is readily accomplished by lotions composed of spirit of ether. A convenient method of keeping up a uniform evaporation, for reducing the temperature of any part of the body, as in the case of fractured limbs, &c., is to allow the gradual distillation of water upon it, through the medium of skeins of cotton, or strips of linen rags, so disposed as to act the part of a syphon, which is readily accomplished by placing one end of the wet cotton in a basin of water, and allowing the other end to hang down over the vessel.

By these means we can directly diminish the activity of the vessels of the part, as, for instance, in burns and scalds, the pain is instantly relieved, and the inflammation reduced: in directing such applications we must take care not so far to reduce the vitality as to endanger the life of the part, and to induce gangrene.

*Internal Refrigerants.*—There are certain saline bodies which, by undergoing a rapid solution, and thus acquiring an increased capacity for caloric, produce a diminution of temperature; and if this take place in the stomach, the sensation of cold which it will produce is equivalent to a partial abstraction of stimulus; which, being extended by sympathy to the heart, occasions a transient reduction in the force of the circulation, and by this, or by a similar sympathetic affection, causes a sensation of cold over the whole body.

The theory advanced to explain the refrigerant operation of vegetable acids, acescent fruits and herbs, and certain other substances, is based on those chemical views respecting animal heat, in which the consumption of oxygen in the act of respiration is considered the principal source. Dr. Murray says,—

"It is established by numerous experiments and observations, that the quantity of oxygen consumed in the lungs is materially influenced by the nature of the ingesta received into the stomach. When the food and drink are composed of substances which contain a small proportion of oxygen, it is known that the consumption of oxygen in the lungs is increased, and this even in a short time after the aliment has been received. Thus Mr. Spelding, the celebrated diver, observed, that whenever he used a diet of animal food, or drank spirituous liquors, he consumed in a much shorter time the oxygen of the atmospheric air in his diving-bell; and he had therefore learnt from experience to confine himself to a vegetable diet, and to water for drink, when following his profession. During digestion, too, it was established by the researches of Lavoisier and Seguin, that a larger proportion of oxygen than usual is consumed. Now, if the animal temperature be derived from the condensation of oxygen gas by respiration, it must follow that an increase in the consumption of that gas will occasion a great evolution of caloric in the system; while a diminution of it will have an opposite effect. If, then, when the temperature of the body is morbidly encreased, we introduce into the stomach substances containing a large proportion of oxygen, especially in a loose state of combination, we may succeed, according to this theory, in reducing the general temperature. This, it is suggested, we may accomplish in part by a vegetable diet, but still more effectually by the free use of the *vegetable acids*, which are readily acted on by the digestive powers, and assimilated with the food; and, as the large quantity of oxygen which they contain is already in a concrete state, little sensible heat can be evolved during the combination of that element with the other principles of the food. The nutritive matter which is received into the blood, containing thus a greater proportion of oxygen than usual, will be disposed to abstract less of it from the air, during its transmission through the lungs, and consequently less caloric will be evolved; the temperature of the body will be reduced; and this again, operating as a reduction of stimulus, will lessen the number and force of the contractions of the heart."

The experiments of Crawford go to prove the chemical origin of animal heat; it is now however generally admitted that the temperature of animals derives its origin from a living principle, although the absorption, in the act of respiration, may indirectly contribute to its production, as a stimulus to the nervous power which produces it. If the heat of the body depended on respiration alone, any one might, by a voluntary effort of quick, deep, and prolonged respiration, increase it at will.

Dr. Murray's theory of animal heat has acquired a renewed interest from the one lately proposed by Professor Liebig, for the purpose of explaining the conversion of the salts of organic acids into carbonates during their transit through the body, and which theory, like that of Dr. Murray's, seeks to establish certain chemical relations between the functions of the digestive and respiratory organs.

"The conversion of these salts of *organic* acids into carbonates," says the Professor, "indicates that a considerable quantity of oxygen must have united with their elements. In order to convert one equivalent of *acetate of potass* into the carbonate of the same base, *eight* equivalents of oxygen must combine with it, of which either *two* or *four* equivalents (according as an acid or neutral salt is produced) remain in combination with the alkali; whilst the remaining six or four equivalents are disengaged as free carbonic acid. There is no evidence presented by the organism itself, to which these salts have been administered, that any of its proper constituents have yielded so great a quantity of oxygen as is necessary for their conversion into carbonates. Their oxidation, therefore, can only be ascribed to the oxygen of the air. During the passage of these salts through the lungs, their acids take part in the peculiar process of *eremacausis* (slow combustion), which proceeds in that organ; a certain quantity of the oxygen gas inspired unites with their constituents, and converts their hydrogen into water, and their carbon into carbonic acid. Part of this latter product (one or two equivalents) remains in combination with the alkaline base, forming a salt which suffers no farther change by the process of oxidation; and it is this salt which is separated by the kidneys or liver. It is then evident, from this theory, that the presence of organic salts in the blood must produce a change in the process of respiration.

"A part of the oxygen inspired, which is usually combined with the constituents of the blood, must, when they are present, combine with their acids, and thus be prevented from performing its usual office. The immediate consequence of which will be the formation of arterial blood in less quantity, or, in other words, the process of respiration must be retarded."

### Antacids.

These are remedies which obviate acidity in the stomach and alimentary canal by combining with the acid and neutralizing it. Here we have a decided instance of chemical action—an acid within the body removed and its effects arrested, by the employment of such chemical substances as are capable of neutralizing an acid out of the body. If the antacid employed be a carbonated alkali or earth, we have a disengagement of carbonic acid, just as we should have in the laboratory, and a new compound formed, the operation of which will vary according to the antacid employed; if magnesia be employed, a slightly purgative salt will be formed; if lime, a salt with an opposite property; the salts formed by the fixed alkalies have a tendency to act on the kidneys. The acid which it becomes necessary to remove may exist in any part of the alimentary canal; very frequently in the cæcum, where vegetable food has been supposed to undergo a second digestion. If the cæcum be the seat of the acid, the fixed alkalies will not be always effectual, as they may be neutralized or absorbed before they can reach the part in question; for which reason magnesia is to be preferred. In some cases, alkaline or saponaceous enemata may be useful to to neutralize acidity in the lower bowels. Under some circumstances *carbonate of ammonia* has an advantage over the fixed alkalies as an antacid, in consequence of its being able to neutralize a portion of the acid matter which appears to exist in a gaseous state in the stomach, and which, on that account, will elude the action of the soda or potass. It is well worth remarking that, where our object is to correct the *lithic acid* diathesis, potass should be preferred to soda, since the latter alkali forms a less soluble salt with the acid in question, and is therefore liable to increase a tendency to urinary deposits.

### Antilithics, and Lithonthryptics.

Antilithics are substances which are considered to possess the power of preventing those mechanical deposits from the urine, which give origin to calculous concretions. They may belong to the class of *vital*, as well as to *chemical* agents.

*Lithonthryptics* are substances such as, by a *chemical* action, have been supposed capable of dissolving, or breaking down calculous concretions, whether lodged in the kidney or bladder. Our author now considers 1st, the composition of healthy urine—2nd, the changes which its several principles undergo in the formation of deposits—3rd, the conditions of the body under which such deposits take place—4th, the remedies by which they may be prevented—and 5th, the means by which, when retained in the body, they may be removed.

1st. *Composition of healthy Urine.*—The quantity voided, and consequently its colour, is necessarily variable, depending on the amount of liquids taken, the condition of the skin, and that of the other excretions, and the state of the atmosphere; but under the ordinary circumstances of health and habits. Dr. Prout considers that, in our climate, the average in 24 hours is from 30 oz. of *specific gravity* 1·025 in the Summer, to 40 oz. of *specific gravity* 1·015 in Winter. There is however a marked distinction between that which is voided after drinking and a moderate meal, and that secreted after the completion of the digestive process; to the former is given the name of *Urina Potûs;* to the latter, and from which alone any correct estimate of its nature can be fairly deduced, that of *Urina Sanguinis.* It should slightly redden litmus paper, and on cooling it very frequently exhibits a slight mucous cloud. In composition this fluid must be considered as the most heterogeneous and variable of all the animal secretions; the proportions of its different ingredients constantly changing, and often without even a reference to any unhealthy condition of the system. The *animal principles* contained in the urine are as follow: water, urea, lithic acid, pure lactic acid, lactate of ammonia, and animal matters not separable from these. Mucus of the bladder. *Alkaline and earthy salts* in the urine: sulphates of potass and of soda—phosphates of soda and of ammonia—muriates of soda and of ammonia—earthy phosphates, with some fluate of lime—silex.

*Urea* formerly was considered as peculiar to urine, and the exclusive result of the action of the kidneys on some constituent of the blood: experiments have now proved that it pre-exists in the blood, and in certain renal diseases is retained by it in considerable quantities, while in health it is immediately removed by the kidneys, those organs, however, merely acting as outlets for its elimination. It is the *Lactic Acid* and the animal matters associated with it that impart to the urine its characteristic odour. Wöhler has proved urea to be a *cyanate of ammonia.* According to Prout urea is a crystallized body, leaving a sensation of coldness on the tongue, like nitre; its smell faint and peculiar, but *not urinous*—it is neither sensibly acid nor alkaline; and although it unites with several acids, thus forming the *nitrate, oxalate* and *lactate of urea,* yet it does not neutralize them. In damp weather it is slightly deliquescent: at 250° it fuses into a colourless fluid, and at a higher temperature is decomposed into *ammonia, cyanate of ammonia,* and *dry solid cyanuric acid.* Combined with the elements of water it assumes the form of *carbonate of ammonia.*

*Lithic Acid.*—With respect to urinary deposits this is by far the most important principle in the urine—it is always combined with ammonia in the natural state of the fluid; from which the weakest acid, however, can separate it. Pour a portion of any acid into urine, and down goes a red precipitate, which is pure *lithic acid.* This is a case of simple affinity; the *lithate of ammonia* is decomposed, the ammonia combines with the precipitant, and the *lithic acid* is separated; upon which plain fact rests the whole theory of lithic acid deposits and the treatment by which they are to be prevented. This acid, like urea, is rich in nitrogen, the former containing 46, the latter 30 per cent.—hence Majendie conceived that its secretion depends on the nitrogen received from alimentary substances, since the urine of animals, confined to food that does not contain it, is free from lithic acid. It does not pre-exist in the blood, but is elaborated by the kidneys, although it sometimes makes its way into the circulation, since the *chalk-stones* deposited in the cellular membrane of gouty persons are composed of *lithate of soda.*

*Alkaline and Earthy Phosphates.*—The phosphoric acid appears to exist in the urine in combination with soda, potass and ammonia, as well as with magnesia and lime; and in such proportions as to constitute super-salts, or biphosphates.

*Lithic acid* appears to be susceptible of various modifications. By the action of dilute nitric acid, with heat, we obtain by gentle evaporation transparent colourless crystals, the *erytheric acid* of Brugnatelli (the alloxan of Liebig.) If, into a strong solution of these crystals whilst boiling hot, we carefully drop some pure ammonia, the solution acquires a beautiful purple colour, and crystals of *purpurate of ammonia* form and subside. If these crystals are treated by means of potass and sulphuric acid, pure *purpuric acid* is obtained in the form of a yellowish or cream-coloured powder. Without entering into further minutiæ, it may be observed that the lithic acid may, through chemical agencies to which the urine is exposed from the reaction of its own elements, be made to assume various characters, by which the aspects of urinary sediments will be influenced: there cannot be a doubt, for instance, that in certain states of disease, the *purpurates* may give to the sediments those beautiful hues formerly attributed by Prout to an acid which he called the *rosacic.*

The *earthy phosphates* may, from an excess in quantity, be separated from the urine, or they may fall down from the action of an alkali abstracting a proportion of phosphoric acid; or a *triple phosphate of magnesia and ammonia* may be produced by the development of ammonia.

The alkaline principles of the urine are often in excess; and it has been observed that an excess of the fixed carbonated alkalies is always accompanied by that of the carbonate of ammonia, a condition of the urine which is highly favorable to a deposition of the phosphates.

*Urea* would appear, under certain circumstances, especially if the fixed alkalies be in excess, to be converted by the kidneys, or occasionally to be decomposed in the bladder, into carbonate of ammonia, which thus becomes the active agent in precipitating the *phosphate of magnesia* in the form of the triple phosphate.

Besides the above-mentioned ingredients of the urine which are essential, we find in certain morbid states *albumen, fibrine, colouring matter of the blood,* which have passed unaltered through the kidneys—*nitric acid—oxalic acid—benzoic acid?—hippuric acid—carbonic acid—xanthic oxide—cystic oxide—sugar—bile, pus* and *mucus.*

*Urinary deposits* may be arranged under three divisions. 1st. *Pulverulent,* or *amorphous sediments.* 2d. *Crystalline sediments,* usually called *gravel.* 3d. *Solid concretions* or *calculi,* formed by the aggregation of the preceding.

1. *Amorphous sediments,* consist of *lithate of ammonia,* or *lithate of soda or of lime,* with colouring matter—or of the *phosphate of lime,* or the *triple phosphate of magnesia and ammonia.* In the latter cases white, or faintly tinged.

2. *Crystalline Deposits.*—When the *lithate of ammonia* is decomposed by a free acid, the lithic acid falls down in small crystals (*red gravel*); whereas the triple phosphate is deposited as white shining crystals, (white gravel.) Sometimes it forms as a pellicle on the surface, exhibiting prismatic colours.

3. *Solid Concretions or Calculi.*—Nitric acid may be habitually discharged for years, before that condition of kidney takes place which appears to dispose its concretion into a calculus. Mr. Earle has endeavoured to shew that the formation of renal calculi may sometimes arise from a local injury affecting the loins and kidney. The lithic acid would appear to be deposited in the *tubuli uriniferi* in a hydrated gelatinous state, and becoming more or less concreted, from the absorption of its water, escapes into the *infundibulum* and *pelvis* of the kidney, from which it may be propelled by the current of urine along the ureter into the bladder.

(We omit giving the arrangement and characters of calculi here, as we gave them in a late number of the Journal.)

III. *The Condition of the System under which Deposits take place, or the Causes by which they are induced.*

The line of demarcation by which healthy and morbid urine are separated from each other is very vague and fugitive, nor are slight deviations of any importance; slight excesses or errors in diet, unusual exercise, or rest, may produce a passing influence on the character of the urine; these Dr. Prout has called the *sediments of health.* The continuance of such sediments, beyond certain limits, might denote a state of the system, which, under the necessary circumstances, might raise them into the importance of gravel or stone.

There are, we have seen, two very distinct species of deposit, the one consisting of lithic acid, the other of phosphoric acid, in combination with different bases. The state of the system in reference to these deposits is as different as the deposits themselves. The lithic acid diathesis always denotes a *sthenic,* while the phosphatic indicates an *asthenic* condition. In the one case the urine is generally acid, in the other it tends to become alkaline. The decomposition of the *lithate of ammonia* takes place from the action of an acid, and it is probable that in the living body the *lactic acid* is the agent by which it is effected, although in some cases we may suppose that it is at once secreted in a free state by the kidney. Now the lactic acid is one of those forms in which Nature expels excrementitious matter from the system, through the skin or kidneys: if therefore it should be abundantly secreted, or its usual exit through the perspiration be obstructed, a preternatural quantity will be thrown upon the kidneys and lithic acid be thus precipitated. Several tropical practitioners have stated that calculous diseases are almost unknown in those latitudes, which almost shows a connexion between the active function of the skin and urinary deposits.

Indigestion is the common cause of the deposition of lithic acid. Unlike the lithic acid deposits, the phosphatic sediments denote a cachectic or asthenic state of the system; alkaline urine is the result of debility. Whatever depresses the powers of life, disposes the urine to become alkaline; hence injuries of the spine are frequently followed by such an effect, as was first noticed by Sir B. Brodie. The transformation of urea into carbonate of ammonia is generally the source from which the phosphate of magnesia derives its ammoniacal precipitant; and this may take place in the bladder in cases where it has lost its *governing power*, as from diseases in the spinal marrow, or from some local affection of the bladder, or prostate gland, in consequence of which the urine being detained, undergoes an incipient process of putrefaction; hence, in elderly people who are frequently incapable of wholly evacuating the bladder, the deposition of the triple phosphate is very common. Alkaline urine is also very deleterious in another respect, by acting as an irritant on the membranous surfaces with which it comes in contact; it sometimes produces chronic inflammation of the mucous membrane, of the kidneys and ureter, extending to that of the bladder.

*Our author next points out the remedial means by which deposits may be prevented.*

Urinary deposits being indications of constitutional disturbance, the first object will be to correct the particular condition of the system on which they depend—it will at the same time be necessary to prevent their deposition in the form of gravel or calculus.

The deposition of the lithates denoting an imperfect performance of the digestive function, the indication will be to restore the assimilative organs to a healthy condition. The decomposition of the lithate, and the consequent deposit of the insoluble lithic acid, constituting *red gravel*, is the effect of an acid re-agent generated in the system. Hence the necessity of avoiding such food as is likely to become acescent; we must also promote a healthy action of the skin by exercise, and if necessary, by the use of vapour baths, by which the system will be relieved from the acid matter which must otherwise pass off by the urine. The anti-lithic medicines to be employed are the alkalies, and alkaline earths, and especially magnesia. Potass is to be preferred to soda. Carbonate of magnesia, in doses of ℈j. to ʒj. is a valuable substitute for alkalies in the lithic acid diathesis. Great caution is necessary in the exhibition of alkaline remedies. Healthy urine will turn blue litmus paper a little red, and alkalies should not be employed to such an extent as to destroy this property; still less ought it to be rendered alkaline; it should not, for instance, be made capable of restoring the blue colour of litmus. Lime-water has been recommended as an antacid. The common saline draught is often found an agreeable mode of giving an alkaline anti-lithic. The occasional interposition of an aperient during an alterative course will be found useful.

Dr. Ure has recently ascertained that benzoic acid has the property of converting the lithic into hippuric acid. If this be true, we are presented with an agent which may be very usefully applied. Dr. Ure states that, either the benzoic acid itself, or a benzoic salt will fulfil the desired indication, the dose being regulated according to the state of the renal secretion—he recommends it to be combined with the *phosphate* or *biborate of soda*, either of which salts will increase its solubility without diminishing its specific power.

In endeavouring to check the deposition of the *phosphates*, we should remember that we have to deal with a state of the system very different from that which exists in the *lithic acid* diathesis. The *phosphates* being deposited by an alkaline re-action of the urine, it is evident that acids are the appropriate anti-lithics—great caution however is necessary here in prescribing either an acid or alkali; for whenever that natural acidity of the urine is destroyed which is necessary for keeping the phosphoric salts in solution, we shall produce a sabulous deposit. Where an acid is indicated the *hydrochloric* or *nitric* are as effectual as any that can be given.

With respect to the means by which calculous deposits, retained in the body, may be removed, there is considerable difficulty, in as much as the resistance caused by the cohesive power and the small surface which they present to the decomposing agent, render such a concentration of chemical power necessary for their solution, as neither the stomach nor bladder could bear with impunity. However, though we may not succeed in materially affecting large concretions, we may make an impression on small calculi, so as to blunt their sharp edges, and cause them to be discharged by the urethra with less difficulty; we may even convert a rough into a smooth calculus, and we may prevent its further increase by modifying the character of the urine, but great care and judgment are necessary to accomplish this. If a lithic acid calculus be present in the bladder, its increase may be prevented by alkalies, but should these be carried too far, we shall cause a deposit of the phosphates.

The mineral acids seem to exert a greater solvent influence on phosphatic calculi, than the alkalies do on those of the lithic acid. The injection of dilute nitric acid through the urethra into the bladder has been tried by Sir B. Brodie and has proved successful. This method is peculiarly deserving attention, as being an application in cases in which neither lithotomy, nor lithotrity can be employed with safety or success, viz. in cases of the deposition of the phosphates, accompanied with a diseased state of the prostate and bladder.

We find we have gone so far in our analysis of this work that we must stop short here. The author we find has made several emendations in this edition which must considerably enhance its value—he has done more; he has incorporated several of Liebig's new views on the actions of substances taken into the system. His remarks on the action of diuretic medicines, on the most judicious and appropriate method of selecting them so as to suit the particular case, are invaluable to the practitioner; indeed this one section leaves Dr. Paris's book without a rival. The section on Antilithics and Lithonthryptics contains valuable rules and directions well deserving careful and attentive perusal. The subject of Urinary Diseases is treated with peculiar clearness and great judgment; in fact this part of the work may be considered an excellent analysis of the best works we possess on this important class of diseases; more especially of the works of Sir B. Brodie and Dr. Prout—in short we may sum up our opinion of Dr. Paris's *ninth* edition of his Pharmacologia in three words; *decies repetita placebit.*

Before quitting the Doctor, however, we have one word to say to him regarding his Greek—at page 87, he gives in a note a passage from the Hecuba of Euripides, Scil. *ναςμος μελαναυγης*, not *μελαναυγες*, as the Doctor has it, and he favours his readers with a translation of it; he renders it, *black-flowing splendour*—surely the least reflexion would have shown him that to such words no rational idea can be annexed—*black-flowing splendour* is decided nonsense—and we assure Dr. Paris that it is not " all the same in the Greek"—the obvious and simple meaning of the words is, *black-shining stream.* It is astonishing that this error pervades all the former editions. We apprehend *Dulces reminiscitur Argos* will not be predicated of Dr. Paris after such a specimen.

